# Supplementary material for: Content-rich biological network constructed by mining PubMed abstracts
Source: BMC Bioinformatics. 2004 Oct 8;5:147. doi: 10.1186/1471-2105-5-147 (PMC528731; doi:10.1186/1471-2105-5-147)
Supplement: Additional File 2 — The original results of the above study (non-essential files are deleted to keep the file size under the limit set by BMC bioinformatics). [file 1471-2105-5-147-S2.bz2 › chilibotAdditionalFile2/dip10/495ID10187774E10658/html/ACVRL1_TGFBR2.html]

 


 **ACVRL1** and **TGFBR2** 
  
Found 11 abstracts in PubMed, retrieved 10.  
 

 What does Google say? 
 PDF only 
| .edu only 

---

**Interactive relationship** (e.g. stimulation, inhibition, etc)

**Non-interactive relationship** (e.g. studied together, co-existance, homology, etc.)

- We demonstrate that primary cultured human thymic epithelial cells TEC express TGF beta 1, TGF beta 2 and TGF beta 3, as well as TGF beta type I receptor T beta RI ALK  [ **ACVRL1** ]  5 and TGF beta type II receptor  [ **TGFBR2** ]  T beta RII transcripts.  Ref: 8562514 Int Immunol, 1995
- Eighty two % of these pancreatic cancers had genetic inactivation of the DPC4, p15, ALK  [ **ACVRL1** ]  5, or  **TGFBR2**  genes.  Ref: 9850059 Cancer Res, 1998
- The blood vessel defects in ALK1  [ **ACVRL1** ]  deficient mice are reminiscent of mice lacking TGF beta1, TGF beta type II receptor  [ **TGFBR2** ]  TbetaR II, or endoglin, suggesting that ALK1  [ **ACVRL1** ]  may mediate TGF beta1 signal in endothelial cells.  Ref: 10716993 Proc Natl Acad Sci U S A, 2000
- We therefore studied the expression of UPA, UPAR, IGF2R, ALK5,  **TGFBR2** , TGFBR3, ENG, ALK1  [ **ACVRL1** ] , TGFB1, TGFB2, and TGFB3 in a series of 14 pancreatic carcinoma cell lines.  Ref: 11408925 Int J Oncol, 2001
